# Ultrafine MoP Nanoparticle Splotched Nitrogen‐Doped Carbon Nanosheets Enabling High‐Performance 3D‐Printed Potassium‐Ion Hybrid Capacitors

**DOI:** 10.1002/advs.202004142

**Published:** 2021-02-02

**Authors:** Wei Zong, Ningbo Chui, Zhihong Tian, Yuying Li, Chao Yang, Dewei Rao, Wei Wang, Jiajia Huang, Jingtao Wang, Feili Lai, Tianxi Liu

**Affiliations:** ^1^ School of Chemical Engineering Zhengzhou University Zhengzhou 450001 P. R. China; ^2^ State Key Laboratory for Modification of Chemical Fibers and Polymer Materials College of Materials Science and Engineering Innovation Center for Textile Science and Technology Donghua University Shanghai 201620 P. R. China; ^3^ Institute of Materials Science and Technology Technische Universität Berlin Straße des 17. Juni Berlin 10623 Germany; ^4^ School of Materials Science and Engineering Jiangsu University Zhenjiang 212013 P. R. China; ^5^ Beijing Key Laboratory of Bio‐inspired Energy Materials and Devices School of Space and Environment Beihang University Beijing 100191 P. R. China; ^6^ Department of Chemistry KU Leuven Celestijnenlaan 200F Leuven 3001 Belgium

**Keywords:** 3D printing, hybrid capacitors, hydrogels, MoP, potassium‐ion storage

## Abstract

Size engineering is deemed to be an adoptable method to boost the electrochemical properties of potassium‐ion storage; however, it remains a critical challenge to significantly reduce the nanoparticle size without compromising the uniformity. In this work, a series of MoP nanoparticle splotched nitrogen‐doped carbon nanosheets (MoP@NC) is synthesized. Due to the coordinate and hydrogen bonds in the water‐soluble polyacrylamide hydrogel, MoP is uniformly confined in a 3D porous NC to form ultrafine nanoparticles which facilitate the extreme exposure of abundant three‐phase boundaries (MoP, NC, and electrolyte) for ionic binding and storage. Consequently, MoP@NC‐1 delivers an excellent capacity performance (256.1 mAh g^−1^ at 0.1 A g^−1^) and long‐term cycling durability (89.9% capacitance retention after 800 cycles). It is further confirmed via density functional theory calculations that the smaller the MoP nanoparticle, the larger the three‐phase boundary achieved for favoring competitive binding energy toward potassium ions. Finally, MoP@NC‐1 is applied as highly electroactive additive for 3D printing ink to fabricate 3D‐printed potassium‐ion hybrid capacitors, which delivers high gravimetric energy/power density of 69.7 Wh kg^−1^/2041.6 W kg^−1^, as well as favorable areal energy/power density of 0.34 mWh cm^−2^/9.97 mW cm^−2^.

## Graphical abstract

Due to the usage of water‐soluble polyacrylamide as molecular skeleton and the strong chemical bond connection inside the hydrogel network, ultrafine MoP nanoparticles can be formed and evenly confined in 3D porous nitrogen‐doped carbon (NC) framework. This can create abundant three‐phase boundaries for efficient response between MoP, NC, and electrolyte, endowing high energy/power 3D‐printed potassium‐ion hybrid capacitors.

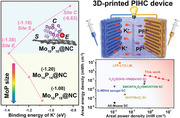

## 1 Introduction

Since the highly increasing dependence on scarce fossil energy, the energy crisis is emerging by accompanying serious environmental pollution, which makes the demand of affordable and green energy storage systems an urgent issue.^[^ ^1^ ^]^ Owing to the high energy density, lithium‐ion batteries (LIBs) are widely used in various portable electronic devices and electric vehicles.^[^ ^2^ ^]^ Nonetheless, the uneven distribution and rising cost of lithium resources have greatly impeded the large‐scale applications of LIBs. Considering the abundant reserve of potassium resources and similar redox potential of K/K^+^ (−2.93 V vs standard hydrogen electrode) to Li/Li^+^ (−3.04 V vs standard hydrogen electrode), potassium‐ion batteries (PIBs) serve as a promising substitute for LIBs to provide a high‐voltage platform and energy density.^[^ ^3^ ^]^ In general, the main drawback of PIBs is their poor energy output rate which is expected to be addressed by constructing potassium‐ion hybrid capacitors (PIHCs) with the synergistic effect of batteries and supercapacitors.^[^ ^4^ ^]^ In detail, the PIHCs device is consisted of capacitor‐type cathode with fast charge/discharge capability and battery‐type anode with high capacity value, resulting in satisfactory potassium‐ion storage properties and energy output rates.

Recently, transition metal phosphides (TMPs) have attracted attention as electrode materials for potassium‐ion storage owing to their high electronic conductivity and electrochemical activity.^[^ ^4^, ^5^ ^]^ However, as observed in the vast majority of metallic compounds (such as MoSe_2_,^[^ ^3a^ ^]^ Nb_2_O_5_,^[^ ^6a^ ^]^ and NiS_2_^[^ ^6b^ ^]^), TMPs‐based potassium‐ion storage systems also suffer from significant volume expansion and sluggish redox kinetics during potassiation/depotassiation process because of the larger radius of potassium ions (1.38 Å vs 0.76 Å for lithium ions).^[^ ^3^, ^7^ ^]^ The surface‐anchoring strategy is regarded as an effective method which disperses TMP nanoparticles on the surface of the conductive carbonaceous matrix (e.g., graphene, carbon nanotubes, and carbon nanofibers), resulting in abundant optimized channels for K^+^ diffusion and electron transfer.^[^ ^4^, ^8^ ^]^ However, the surface‐anchoring strategy can only slightly withstand the volume change during the potassiation/depotassiation process owing to a partial surface exposed to the electrolyte, resulting in poor cycling stability for practical applications. Hence, polymer gels with interconnected nanostructures could be potentially used as precursors for hierarchical carbon skeletons which serve as supports for homogenously encapsulating TMP nanoparticles and provide open mass/charge transport pathways. In addition, polymer gels could effectively control the location, size, and density of TMP species at the molecular level and offer a promising platform to investigate the relationship between the size effect of TMP nanoparticles and electrochemical properties.^[^ ^9^, ^10^ ^]^ As a conventional water‐soluble polymer, polyacrylamide (PAM) can be easily mixed with the precursors of TMPs to form a homogenous hydrogel which can realize the uniform dispersion of ultrafine TMP nanoparticles inside an interconnected carbon converted from the PAM‐based hydrogel.^[^ ^11^ ^]^ Coincidentally, the abundant amino groups (‐NH_2_) in PAM chains could serve as a self‐carrying nitrogen source to achieve homogenous nitrogen doping in the carbon matrix and possibly improve its bonding effect toward potassium ions.^[^ ^3^, ^11^ ^]^

In addition to the numerous efforts on the development of electrode materials, the design of advanced electrodes with customized architectures plays a crucial role in elevating the energy storage performance and increasing possible practical applications. Owing to the advances in additive manufacturing techniques (e.g., excellent process flexibility and good geometry controllability), extrusion‐based 3D printing is an emerging technology for fabricating multifarious devices in the field of energy storage.^[^ ^12^ ^]^ In particular, direct ink writing (DIW) is among the most commonly used 3D‐printed techniques to readily achieve complex geometric shapes that are difficult to generate using conventional methods. Moreover, owing to the mechanically stable architectures and macroscopic interconnected networks, the DIW‐printed electrodes exhibit adequate charge transport channels to achieve excellent electrochemical performance even at a high mass loading. Although certain preliminary outcomes have been achieved using 3D‐printing methods, such as supercapacitors,^[^ ^13a,13b^ ^]^ lithium‐ion batteries,^[^ ^13c^ ^]^ and lithium–sulfur batteries,^[^ ^13d^ ^]^ achieving good compatibility for the multicomponent inks and desirable power/energy density for a completely 3D‐printed PIHCs device (3DP‐PIHCs) remains a challenge.

Herein, we explore a novel method by anchoring ultrafine MoP nanoparticles in PAM hydrogel‐derived carbon nanosheets to form MoP nanoparticles with nitrogen‐doped carbon nanosheets (MoP@NC). Owing to the use of water‐soluble PAM hydrogel with strong chemical bonds in the molecular skeleton, ultrafine MoP nanoparticles could be confined to uniformly generate in 3D and porous NC. This helps to reduce the volume expansion of the MoP phase during the charge/discharge process and provide conductive pathways for K^+^ diffusion and electron transfer. Consequently, the MoP@NC‐1 electrode delivers an excellent capacity performance (256.1 mAh g^−1^ at 0.1 A g^−1^) and a long‐term cycling durability (89.9% capacitance retention after 800 cycles). As observed from density functional theory (DFT) calculations, the abundant three‐phase boundaries (ultrafine MoP nanoparticles, NC networks, and electrolyte) could shift their binding energy toward potassium ions from −1.08 eV (Mo_18_P_18_@NC model) to −1.38 eV (Mo_10_P_10_@NC model). Furthermore, we applied the MoP@NC‐1 as an additive for 3D‐printing ink to fabricate 3D‐printed potassium‐ion hybrid capacitors (3DP‐PIHCs), which delivers not only high gravimetric energy/power density of 69.7 Wh kg^−1^/2041.6 W kg^−1^ but also favorable areal energy/power density of 0.34 mWh cm^−2^/9.97 mW cm^−2^.

## 2 Results and Discussion

**Scheme 1:**
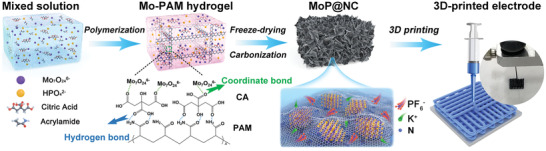
Schematic illustration for the synthetic procedure of MoP@NC composites and 3D‐printed electrode (inset at the top‐right corner: photograph of 3D‐printed electrode).

The synthetic process of MoP nanoparticles splotched nitrogen‐doped carbon nanosheets (MoP@NC) is illustrated in **Scheme** **1**. Here, (NH_4_)_6_Mo_7_O_24_·4H_2_O, (NH_4_)_2_HPO_4_, and citric acid (CA) were mixed to generate a homogenous solution. Then, acrylamide was added to initiate the free‐radical polymerization and form a Mo‐polyacrylamide (Mo‐PAM) hydrogel. Here, Mo_7_O_24_^6−^ was uniformly dispersed on PAM chains using coordinate bonds between Mo_7_O_24_^6−^ and CA as well as hydrogen bonds between CA and PAM, leading to the formation of 3D networks in Mo‐PAM hydrogel. Subsequently, the Mo‐PAM hydrogel could be changed into a MoP@NC composite after freeze‐drying treatment and carbonization under a H_2_/Ar atmosphere. Additionally, we observed that the size of the MoP nanoparticles could be appropriately modulated by controlling the heating rate during carbonization (1, 5, and 10 °C min^−1^), which are denoted as MoP@NC‐1, MoP@NC‐2, and MoP@NC‐3, respectively (further details are found in the Supporting Information). Because of the abundant amino groups in the PAM chains, they could be regarded as a self‐carrying nitrogen source to achieve uniform nitrogen doping in the carbon matrix. Consequently, the MoP@NC‐based ink was obtained by homogenously mixing MoP@NC, graphite oxide (GO), and carbon nanotube (CNT) suspension at a certain concentration (further details are in the Supporting Information) which was loaded into a program‐controlled 3D printer to produce customized electrodes on any substrate. As shown in the digital photos in Scheme 1 and Figures S1 and S2 in the Supporting Information, the 3D‐printed electrode on the poly(tetrafluoroethylene) substrate exhibits excellent mechanical stability and versatility.

**Figure 1:**
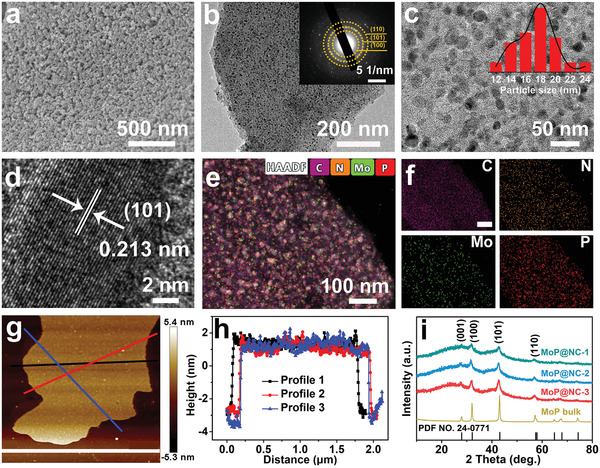
a) SEM image of MoP@NC‐1. b) Low‐magnified TEM image of MoP@NC‐1 (inset: SAED pattern). c) High‐magnified TEM image (inset: particle size distribution of MoP in MoP@NC‐1), d) HRTEM image, e) high‐angle annular dark‐field scanning transmission electron microscopy image, and f) corresponding element mappings for C, N, Mo, and P elements of MoP@NC‐1 (the scale bar is 100 nm). g) AFM image and (h) the corresponding height profiles of MoP@NC‐1. i) X‐ray diffraction patterns of various MoP@NC composites and MoP bulk.

The scanning electron microscopy (SEM) image in **Figure** **1**a shows that MoP@NC‐1 exhibits an open and porous structure which could provide a highly exposed surface owing to its good contact with the electrolyte. With an increase in the heating rate during carbonization, the MoP nanoparticles would easily agglomerate into larger nanoparticles, where partial pores in MoP@NC‐2 are blocked (Figure S3a, Supporting Information) and large MoP particles appear on the surface of MoP@NC‐3 (Figure S3b, Supporting Information). This is because MoP nanoparticles tend to agglomerate at a higher heating rate.^[^ ^14^ ^]^ The 3D network of MoP@NC‐1 is composed of abundant 2D nitrogen‐doped carbon nanosheets. The transmission electron microscopy (TEM) images of individual MoP@NC‐1 nanosheets (Figure 1b,c) show the ultrafine MoP nanoparticles splotched on porous NC skeleton by forming abundant two‐phase boundaries (MoP nanoparticles and NC). The selected area electron diffraction (SAED) pattern confirms the polycrystalline characteristics of the MoP phase (inset of Figure 1b). Size distribution quantification of MoP nanoparticles in MoP@NC‐1 is provided in the corresponding histograms with an average particle size of around 17.5 nm (inset of Figure 1c). Owing to the uniform dispersion of the MoP precursor in the PAM hydrogel and confined effect from the NC skeleton, the severe agglomeration of MoP bulk (Figure S4, Supporting Information) can be avoided. The high‐resolution TEM (HRTEM) image of MoP@NC‐1 (Figure 1d) displays the lattice space of the MoP component measured at 0.213 nm from a vertical view, which corresponds to the (101) plane of the MoP phase and demonstrates the (101) plane is the preferred orientation in MoP@NC‐1.^[^ ^2a^ ^]^ The energy‐dispersive X‐ray spectrometry mappings (Figure 1e,f) were conducted to determine the distribution of C, N, Mo, and P elements in MoP@NC‐1. The element distributions of Mo and P are highly overlapped due to the MoP phase, while the homogenous appearance of N proves nitrogen doping in the NC network. To investigate the thickness of the MoP@NC‐1 nanosheets, atomic force microscopy (AFM) was conducted in different directions (Figure 1g). Its results demonstrate an extra‐low thickness of ≈5 nm (Figure 1h). The X‐ray diffraction patterns in Figure 1i show distinct broad diffraction peaks at ≈24° in the MoP@NC composites and NC (Figure S5, Supporting Information). The peaks correspond to the (002) plane of the amorphous carbon structure.^[^ ^3^, ^15^ ^]^ The characteristic peaks observed at 27.9°, 32.3°, 43.1°, and 57.4° correspond to the (001), (100), (101), and (110) planes of hexagonal MoP phase, respectively (JCPDS No. 24–0771), which indicates the successful preparation of MoP@NC composites.

**Figure 2:**
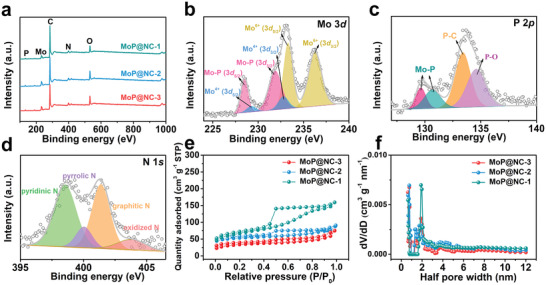
a) XPS survey spectra of various MoP@NC composites. High‐resolution XPS spectra for b) Mo 3d, c) P 2p, and d) N 1s of MoP@NC‐1. e) N_2_ adsorption–desorption isotherms measured at −196 °C and f) pore size distribution plots of various MoP@NC composites.

X‐ray photoelectron spectroscopy (XPS) measurements were conducted to analyze the chemical composition and state of the MoP@NC composites. The XPS survey spectra of various MoP@NC composites (**Figure** **2**a) show the principal signals of C, N, Mo, and P elements. In the high‐resolution Mo 3d XPS spectra of MoP@NC‐1 (Figure 2b), two peaks located at 228.5 and 231.8 eV could be attributed to Mo 3d_5/2_ and 3d_3/2_, respectively, in the MoP phase. In addition, the other two doublets at 229.2/232.8 and 233.3/236.3 eV originate from Mo^4+^ in MoO_2_ and Mo^6+^ in MoO_3_, respectively.^[^ ^16^ ^]^ The high‐resolution P 2p spectra of MoP@NC‐1 in Figure 2c show the peaks at 129.6 and 130.6 eV that are attributed to the Mo−P species in MoP.^[^ ^8^, ^17^ ^]^ The peaks located at 133.3 and 134.5 eV originate from the P—C and P—O bonds, respectively, indicating a strong interaction between the MoP and NC network.^[^ ^18^ ^]^ The high‐resolution N 1s spectra of MoP@NC‐1 (Figure 2d) indicate the existence of four types of nitrogen species: pyridinic‐type nitrogen (398.4 eV), pyrrolic‐type nitrogen (399.8 eV), graphitic‐like type nitrogen (401.3 eV), and pyridine‐N‐oxides (404.2 eV).^[^ ^11a^ ^]^ Nitrogen (−196 °C) adsorption–desorption isotherms for various MoP@NC composites and MoP bulk were investigated to determine their specific surface area (SSA) values and corresponding pore structures (Figure 2e,f and Figure S7, Supporting Information). As observed from the type IV characteristic curves with H1 hysteresis loops in Figure 2e, there are abundant mesoporous structures in various MoP@NC composites which are further confirmed by their corresponding pore size distributions (Figure 2f). The SSA value of MoP@NC‐1 (229.3 m^2^ g^−1^) is much higher than those of MoP@NC‐2 (167.8 m^2^ g^−1^), MoP@NC‐3 (122.9 m^2^ g^−1^), and MoP bulk (4.6 m^2^ g^−1^). The highest SSA value of MoP@NC‐1 is because the carbon pores could be completely divided by the ultrafine MoP nanoparticles due to the generation of abundant rough hole walls. This would result in a new surface and increased SSA value.^[^ ^19^ ^]^

**Figure 3:**
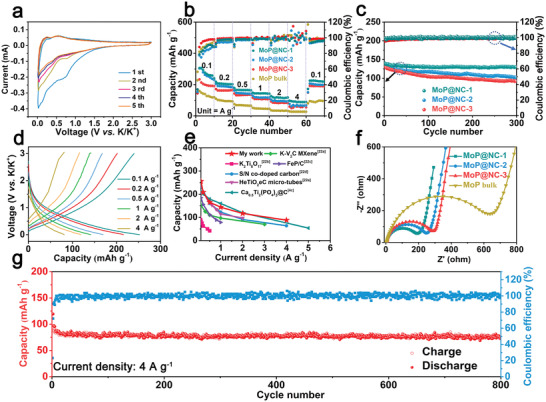
a) CV curves of the MoP@NC‐1 in the first five cycles at a scan rate of 0.1 mV s^−1^. b) Rate capability of various MoP@NC composites and MoP bulk. c) Cycling performance of various MoP@NC composites at 1 A g^−1^. d) GCD profiles of MoP@NC‐1 at various current densities. e) Comparison of the potassium‐ion storage performance of MoP@NC‐1 with previously reported materials for potassium‐ion storage. f) Nyquist plots of various MoP@NC composites and MoP bulk. g) Cycling performance of MoP@NC‐1 at a high current density of 4 A g^−1^.

Considering the evolution of the MoP nanoparticle size, the relationship between the size effect and electrochemical performance was investigated in half‐battery constructions. The electrochemical properties of the MoP@NC‐1 were first characterized using cyclic voltammetry (CV) in the potential range of 0.01−3.0 V versus K/K^+^ at a sweep rate of 0.1 mV s^−1^ (**Figure** **3**a). In the first cycle, a cathodic peak was observed at ≈0.75 V with a high current response. This can be ascribed to the formation of a solid electrolyte interface which is an irreversible reaction derived from the phase change on the electrode in the first cathodic sweep.^[^ ^20^ ^]^ Moreover, the overlapped CV curves of the following cycles reveal the excellent electrochemical reversibility of the MoP@NC‐1 anode during the potassium‐ion storage process.^[^ ^21^ ^]^ Figure 3b shows the rate capability of various MoP@NC composites and MoP bulk at different scan rates from 0.1 to 4 A g^−1^. The initial Coulombic efficiency for MoP@NC‐1/2/3 and MoP is 32.8%, 32.5%, 24.5%, and 32.5% at 0.1 A g^−1^, respectively. For MoP@NC‐1, reversible capacities of 256.1, 203.2, 165.3, 143.8, and 116.8 mAh g^−1^ can be obtained at current densities of 0.1, 0.2, 0.5, 1, and 2 A g^−1^, respectively. It maintains a reversible capacity of 89.3 mAh g^−1^ even at a high current density of 4 A g^−1^. When the current density returned to 0.1 A g^−1^ after 50 cycles, the reversible capacity recovered to 236.7 mAh g^−1^, thus demonstrating its excellent rate performance. For comparison, the reversible capacities of MoP@NC‐2, MoP@NC‐3, and MoP anodes were reduced rapidly to 69.4, 54.7, and 26.7 mAh g^−1^ at 4 A g^−1^, respectively. Benefiting from interconnected pathways for electronic transfer derived from the 3D porous NC framework, the MoP@NC composites show an improved rate capacity when compared with MoP bulk. In addition, based on the equation *τ* ≈ *L* ^2^/*D*, *τ* (ionic diffusion time) is proportional to the square of *L* (ionic diffusion length). Thus, the enhanced rate capability of MoP@NC‐1 with reduced MoP nanoparticle size may be attributed to the shorter migration path of the potassium ion. Figure 3c presents the cycling stabilities of various MoP@NC composites at 1 A g^−1^, from which MoP@NC‐1 anode displays an improved capacitance retention of 95.1% after 300 cycle with a stable Coulombic efficiency as compared to those of MoP@NC‐2 (77.7%) and MoP@NC‐3 (70.3%). According to the galvanostatic charging/discharging (GCD) curves of MoP@NC‐1 at different current rates (Figure 3d), the almost overlapping profiles imply the favorable reversibility of the potassium‐ion storage behavior. The delicately designed MoP@NC‐1 exhibits further outstanding or comparable rate capability when compared with other electrode materials for potassium‐ion storage (Figure 3e).^[^ ^4^, ^22^ ^]^ The enhanced potassium‐ion storage performance of MoP@NC‐1 is attributed to the synergistic effect between the MoP nanoparticles and NC networks. The uniformly formed coordinate bonds between Mo_7_O_24_^6−^ and CA facilitate further easy uniform dispersion of MoP precursors. This results in the generation of a porous NC skeleton and ultrafine MoP nanoparticles (Figure 1c) with abundant two‐phase boundaries (MoP nanoparticles and NC) which is beneficial for the diffusion of KPF_6_ electrolyte and insertion/extraction of potassium ions.^[^ ^4^, ^8^ ^]^ Additionally, electrochemical impedance spectroscopy analysis was conducted, from which two parts (i.e., a depressed semicircle in the high‐medium region and an inclined line in the low‐frequency region) could be observed for various MoP@NC composites and MoP bulk (Figure 3f and Figure S9, Supporting Information). The charge‐transfer resistance (*R*_ct_) of MoP@NC‐1 (*R*_ct_ = 192.9 Ω) is much lower than those of MoP@NC‐2 (*R*_ct_ = 249.2 Ω), MoP@NC‐3 (*R*_ct_ = 290.7 Ω), and MoP bulk (*R*_ct_ = 642.3 Ω). This is because the larger nanoparticles among MoP@NC‐2, MoP@NC‐3, and MoP bulk deepened the insufficient infiltration effect along with the confined electrons and ion transfer. This indicates that the regulation of nanoparticle size is important to improve the interface resistance. For the investigation of practical applications under high current density, Figure 3g displays the cycling performance of MoP@NC‐1 at 4 A g^−1^. MoP@NC‐1 shows a stable cycling lifespan over 800 cycles with a high capacity retention of 89.9% and Coulombic efficiency of ≈100%.

**Figure 4:**
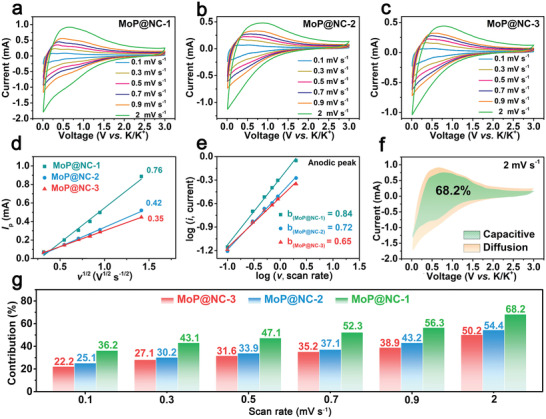
The CV curves at different scan rates (0.1, 0.3, 0.5, 0.7, 0.9, and 2 mV s^−1^) for a) MoP@NC‐1, b) MoP@NC‐2, and c) MoP@NC‐3. d) The linear relation between *I*_p_ and *v* ^1/2^, and e) the relation between log(*i*) and log(*v*) of various MoP@NC composites. f) The capacitive contribution (green) and the diffusion contribution (orange) of CV profile of MoP@NC‐1 at a scan rate of 2 mV s^−1^. g) Normalized proportions for capacitive contributions of various MoP@NC composites.

To further demonstrate the relationship between the MoP nanoparticle size and kinetic behavior, the CV curves of various MoP@NC composites were analyzed at different scan rates from 0.1 to 2 mV s^−1^ (**Figure** **4**a–c). When comparing the CV curves of various MoP@NC composites, the reduction peak of MoP@NC‐1 is enlarged and shows a larger area among the three samples (Figure S11, Supporting Information). This is because similar storage sites theoretically require similar energy for participating in the related redox reaction. Thus, the redox peaks would be broadened if storage sites possess an extensive distribution of energy level. The reduced MoP nanoparticle sizes in MoP@NC‐1 could expose extra surface of crystalline grains and induce additional active sites of different energy levels favoring fast K^+^ insertion/extraction processes, particularly at high‐rate densities.^[^ ^21^ ^]^ Furthermore, Figure 4d shows the linear relationship between *I_p_* and *v* ^1/2^ of various MoP@NC composites, where the slope values are 0.76, 0.42, and 0.35 for MoP@NC‐1, MoP@NC‐2, and MoP@NC‐3, respectively. The highest slope value of MoP@NC‐1 proves its enhanced ability for K^+^ transfer during the discharge/charge process because the ultrafine MoP nanoparticles in MoP@NC‐1 can increase the contact area with the electrolyte. As shown in Figure 4e, the relationship between current (*i*) and scan rate (*v*) complies with the power‐law equation *i* = *a^v^b*, where *a* and *b* are adjustable constants. If the calculated *b*‐value equals 0.5, it implies a diffusion‐dominated K^+^ intercalation process, while if *b*‐value approaching 1.0 represents absolute surface‐controlled capacitive behavior.^[^ ^23^ ^]^ Based on the CV profiles and fitting lines of log(*v*) versus log(*i*), the *b*‐values of MoP@NC‐1, MoP@NC‐2, and MoP@NC‐3 were calculated to be 0.84, 0.72, and 0.65, respectively, demonstrating that the electrochemical K^+^ storage process in MoP@NC‐1 is primarily dominated by surface‐controlled capacitive behavior. Moreover, the increasing *b*‐values in the following sequence (MoP@NC‐1 > MoP@NC‐2 > MoP@NC‐3) reveal the enhanced capacitive kinetics in MoP@NC‐1, implying that the reduced nanoparticle size could effectively boost the surface‐controlled capacitive behavior. In particular, based on the equations (*i* = *k*_1_ *v* + *k*_2_ *v*_1/2_ and *i*/*v* ^1/2^ = *k*_1_ *v* ^1/2^ + *k*_2_), the diffusion‐/capacitive‐controlled contribution could be further quantitatively analyzed. The capacitive contributions at different scan rates are further summarized. MoP@NC‐1 achieved the highest capacitive contribution value of 68.2% at 2 mV s^−1^ when compared with 54.4% of MoP@NC‐2 and 50.2% of MoP@NC‐3 (Figure 4f and Figure S12, Supporting Information). As the scan rates increased from 0.1 to 2 mV s^−1^, the capacitive contributions for MoP@NC‐1 gradually increased which are larger than those of MoP@NC‐2 and MoP@NC‐3 at identical scan rates (Figure 4g). More ions would be absorbed on the surface of MoP@NC‐1 at a high scan rate, followed by the surface‐controlled electrochemical behavior. The ultrafine MoP nanoparticle size in MoP@NC‐1 endows more exposed surface area of crystalline grains, resulting in the generation of abundant active boundaries for favored surface‐controlled electrochemical behavior. A detailed reason for the enhanced rate capability of MoP@NC‐1 (Figure 3b) could also be related to its enhanced surface‐controlled behavior.

**Figure 5:**
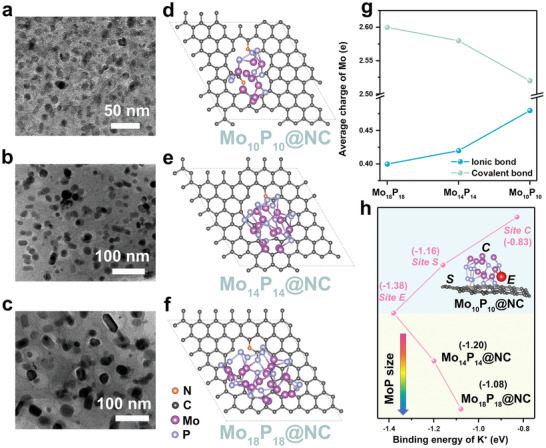
TEM images of MoP@NC with different MoP nanoparticle sizes: a) MoP@NC‐1, b) MoP@NC‐2, c) MoP@NC‐3. Atomic models of MoP@NC with different MoP nanoparticle sizes: d) Mo_10_P_10_@NC, e) Mo_14_P_14_@NC, and f) Mo_18_P_18_@NC. g) Calculated average charges of Mo atom in Mo_18_P_18_, Mo_14_P_14_, and Mo_10_P_10_ models as derived from ionic bond and covalent bond, respectively. h) Calculated K^+^ (red atom) binding energy values on different sites of Mo_10_P_10_@NC, as well as the rims of Mo_14_P_14_@NC and Mo_18_P_18_@NC. The inset is the constructed model for K^+^ binding on the different sites of Mo_10_P_10_@NC, where the indices C, E, and S represent the sites of the center of MoP, rim of MoP and NC, and the surface of exposed NC, respectively.

To comprehend the relationship between the nanoparticle size of MoP and variable potassium‐ion storage performance, DFT calculations were conducted. Based on various morphology of MoP@NC composites and corresponding size distribution quantifications (**Figure** **5**a–c and Figure S13, Supporting Information), three related atomic models with different MoP nanoparticle sizes are given in Figure 5d−f, which are marked as Mo_10_P_10_@NC, Mo_14_P_14_@NC, and Mo_18_P_18_@NC, respectively. As shown in Figure 5g, the covalent bond in MoP nanoparticles was partially transferred into the ionic bond when the MoP nanoparticle size decreased from Mo_18_P_18_ to Mo_10_P_10_. A small MoP domain could present enhanced electron delocalization and generate strong heterojunctions between MoP and carbon. This is regarded as a key issue to enhance the electrolyte adsorption and electronic conductivity of MoP@NC by introducing a strong local electric field at the boundary of MoP nanoparticles and NC networks. Additionally, such increased ionic bonds would help adsorb K^+^ and enhance the specific density of the battery. This implies that smaller the MoP nanoparticle, higher is the activity of MoP@NC. As shown in the constructed Mo_10_P_10_@NC model in the inset of Figure 5h, there are three possible competing sites for the binding of potassium ions: the formal center of MoP nanoparticles (*Site C*), rim of MoP nanoparticles and NC (*Site E*), and non‐MoP‐coated surface of the exposed NC (*Site S*). Subsequently, the specific binding energy values of potassium ions at various sites of the MoP@NC anode were calculated (Figure 5h). The binding energy values are −1.38, −0.83, and −1.16 eV for *Site E*, *Site C*, and *Site S*, respectively; the phase boundary (*Site E*) is demonstrated by the strongest site for potassium‐ion adsorption. In addition, the K^+^ binding energy values at *Site E* of various MoP@NC models (i.e., Mo_10_P_10_@NC, Mo_14_P_14_@NC, and Mo_18_P_18_@NC) are shown in Figure 5h, which are −1.38, −1.20, and −1.08 eV. Thus, this confirms that a smaller MoP nanoparticle size is beneficial to enhance its binding ability toward potassium ions. Further, it reflects the advantages of our synthetic strategy by confining ultrafine MoP nanoparticles in the porous NC network and exposing abundant phase boundary for potassium‐ion storage.

**Figure 6:**
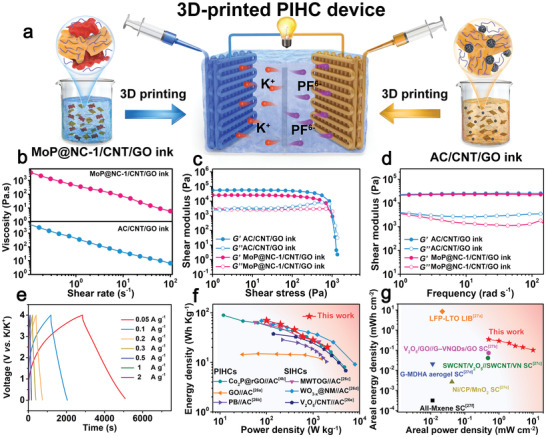
a) Schematic illustration for MoP@NC‐1//AC 3DP‐PIHCs device and corresponding charging process. b) The apparent viscosity as a function of shear rate, c) storage modulus and loss modulus as a function of shear stress, d) dynamic modulus (*G*′ and *G*′′) as a function of frequency for MoP@NC‐1/CNT/GO ink and AC/CNT/GO ink. e) GCD curves of the MoP@NC‐1//AC 3DP‐PIHCs at various current densities. f,g) Ragone plots of the MoP@NC‐1//AC 3DP‐PIHCs in comparison with other reported energy storage devices.

3D‐printing technology has been demonstrated as a burgeoning method which can be used to fabricate PIHC devices with customized architectures through a facile and scalable approach. First, conductive MoP@NC‐1/CNT/GO ink and active carbon (AC)/CNT/GO ink were prepared by mixing active materials (MoP@NC‐1 or AC) with GO and CNT additives in specific mass proportions (further details are found in the Supporting Information). We further assembled 3D‐printed PIHCs (3DP‐PIHCs) using a 3D‐printed MoP@NC‐1 electrode as an anode and 3D‐printed AC electrode as a cathode with a wide working potential range of 0.01−4 V (**Figure** **6**a). Meanwhile, the rheological properties of the prepared inks were measured to ensure the unhindered 3D‐printing process and noncollapsed architecture. As shown in Figure 6b, the MoP@NC‐1/CNT/GO ink and AC/CNT/GO ink possess high apparent viscosities at low shear rates and exhibit non‐Newtonian (shear‐thinning) behaviors, where the apparent viscosities decrease as the shear rates increase. This indicates that the ink would smoothly stream throughout a fine nozzle during the extrusion process. Thus, such properties ensure that the ink can return to a zero‐shear state when the applied stress is eliminated. Hence, the shape of the printed electrodes can be retained to construct a complex framework. Figure 6c shows the storage modulus (*G*′) and loss modulus (*G″*) profiles of MoP@NC‐1/CNT/GO ink as a function of shear stress. Its *G*′ is approximately one magnitude higher than *G″* before shear stress attains 850.6 Pa which represents a conventional elastic‐like solid. When the shear stress exceeded 850.6 Pa, the *G″* value was higher than *G*′, ensuring that the MoP@NC‐1/CNT/GO ink could be extruded smoothly from the needle to the substrate.^[^ ^24^ ^]^ Then, the AC/CNT/GO ink also exhibited characteristics similar to those of the MoP@NC‐1/CNT/GO ink with a yield stress of 933.2 Pa (*G″* > *G*′), indicating the transient point from the solid to liquid state. Additionally, *G*′ is higher than *G″* for the MoP@NC‐1/CNT/GO and AC/CNT/GO inks within the entire frequency range (0.01−100 rads^−1^), thus proving their conventional viscoelastic behaviors at rest (Figure 6d). The rheological behaviors of the MoP@NC‐1/CNT/GO ink and AC/CNT/GO ink implied their good printability for the customized 3DP‐PIHC device without deformation.^[^ ^12^, ^25^ ^]^ The potassium‐ion storage property of the 3DP‐PIHCs device was further evaluated. First, the potassium‐ion storage performance of the 3DP‐AC electrode was evaluated in half‐cells by employing metallic potassium as the counter and reference electrodes. The evaluation was conducted to determine the mass ratio of the MoP@NC‐1 anode to the AC cathode for MoP@NC‐1//AC 3DP‐PIHCs. After considering the working voltage windows of the CV curves for the MoP@NC‐1 and AC electrodes (Figures S14 and S15, Supporting Information), the MoP@NC‐1//AC 3DP‐PIHC was operated with working voltage cut‐off values between 0.01 and 4.0 V. The approximate rectangular shape of the CV curves up to 100 mV s^−1^ (Figure S16, Supporting Information) and symmetric triangle shape of the GCD curves up to 2 A g^−1^ (Figure 6e) indicate that the energy storage process in MoP@NC‐1//AC 3DP‐PIHCs is predominantly dominated by capacitive behavior. Further, MoP@NC‐1//AC 3DP‐PIHCs obtained a high gravimetric energy density of 69.7 Wh kg^−1^ at a gravimetric power density of 2041.6 W kg^−1^ (Figure 6f), which is comparable with certain recently reported sodium/potassium‐ion capacitor devices.^[^ ^4^, ^26^ ^]^ Meanwhile, the MoP@NC‐1//AC 3DP‐PIHCs delivers a high areal energy density of 0.34 mWh cm^−2^ at an areal power density of 9.97 mW cm^−2^ (Figure 6g) which compares favorably with batteries and supercapacitors constructed via printing technology.^[^ ^27^ ^]^ The high energy/power densities and wide potential range of the 3DP‐PIHCs demonstrate that our proposed 3D‐printed electrode design is suitable for future practical applications.

## 3 Conclusion

In summary, we have fabricated 3D‐printed potassium‐ion hybrid capacitors with high energy/power density using a freestanding AC cathode and MoP@NC‐1 anode. The unique confined NC nanoarchitecture with abundantly decorated ultrafine MoP nanoparticles can endow structural compatibility, relieve volume expansion, and provide conductive pathways for K^+^ diffusion and electron transfer. Consequently, the as‐prepared MoP@NC‐1 delivers an improved capacity performance (256.1 mAh g^−1^ at 0.1 A g^−1^) and long‐term cycling durability (89.9% of capacitance retention after 800 cycles). DFT calculations reveal the origin of the size effect for enhanced electrochemical properties. The ultrafine MoP nanoparticles could activate the neighboring areas in NC skeletons to efficiently improve the binding energy toward potassium ions and demonstrate enhanced conductivity with the partial generation of MoP ionic bonds. Furthermore, the thus‐derived 3D‐printed PIHCs device delivered a high gravimetric energy/power density of 69.7 Wh kg^−1^/2041.6 W kg^−1^ and demonstrated a favorable areal energy/power density of 0.34 mWh cm^−2^/9.97 mW cm^−2^. Therefore, this study offers an in‐depth understanding for building a feasible architecture design from microcosmic to macroscopic scale of transition metal phosphide‐based electrodes for potassium‐ion hybrid capacitors.

## Conflict of Interest

The authors declare no conflict of interest.

## Supporting information

Supporting Information
