# Supplementary material for: Ultrafine MoP Nanoparticle Splotched Nitrogen‐Doped Carbon Nanosheets Enabling High‐Performance 3D‐Printed Potassium‐Ion Hybrid Capacitors
Source: Adv Sci (Weinh). 2021 Feb 2;8(7):2004142. doi: 10.1002/advs.202004142 (PMC8025015; doi:10.1002/advs.202004142)
Supplement: Supplementary file 1 — Supporting Information [file ADVS-8-2004142-s001.pdf]

© 2021 Wiley-VCH GmbH

ADVANCED SCIENCE
Open Access

Supporting Information

for *Adv. Sci.,* DOI: 10.1002/advs.202004142

Ultrafine MoP Nanoparticles Splotched Nitrogen-Doped Carbon Nanosheets Enabling High-Performance 3D-Printed Potassium-Ion Hybrid Capacitors

Wei Zong,† Ningbo Chui,† Zhihong Tian, Yuying Li, Chao Yang, Dewei Rao, Wei Wang, Jiajia Huang, Jingtao Wang, Feili Lai,* Tianxi Liu*

Supporting Information

Ultrafine MoP Nanoparticles Splotched Nitrogen-Doped Carbon Nanosheets Enabling High-Performance 3D-Printed Potassium-Ion Hybrid Capacitors

Wei Zong,[†] *Ningbo Chui,*[†] *Zhihong Tian, Yuying Li, Chao Yang, Dewei Rao, Wei Wang, Jiajia Huang,*[*] *Jingtao Wang, Feili Lai,*[*] *Tianxi Liu*

[†]W. Zong and N. Chui contributed equally to this work.

[*]Corresponding author e-mail: huangjiajia@zzu.edu.cn; feili.lai@kuleuven.be

Experimental

1. Materials Synthesis and Characterization:

Preparation of the MoP@NC. Typically, 0.353 g of $(NH_4)_6Mo_7O_{24}\cdot4H_2O$, 0.768 g of citric acid (CA), 0.264 g of $(NH_4)_2HPO_4$, and 20 mL deionized (DI) water were added into a 50 ml three-necked flask, which was kept and stirred at 85 °C for 20 h. After cooling to room temperature, 1.5 g of acrylamide (AM) and 0.27 g of *N, N'*-methylenebis (acrylamide) (MBA) were added into the above solution under a low-pressure nitrogen atmosphere. After stirring for 20 minutes, 0.5 g of $ZnCl_2$ and 0.5 g of NaCl were dissolved to form a clear and colorless solution. Subsequently, 1 mL aqueous solution, containing 40 μL of *N, N, N', N'*-Tetramethylethylenediamine (TEMED) and 75 mg of ammonium persulfate (APS), was slowly added into it continuously to initiate the free-radical polymerization at 65 °C and keep for 5 h. The precursors of MoP@NC were got after the as-produced hydrogel was cut into small pieces and changed into aerogels after freeze-drying treatment. Then, the aerogels were maintained at 300 °C for 1 h and carbonized at 950 °C for 2 h (heating rate: 1 °C min^{-1}) under the protection of a H_2/Ar atmosphere (volume ratio: 1:9). Finally, the MoP@NC-1 was washed with dilute HCl solution, DI water and ethanol in turn, and dried in an oven at 60 °C overnight. Additionally, another two composites were also prepared with the same synthetic steps, while the heating rate for MoP@NC-2 and MoP@NC-3 composites were 5 °C min^{-1} and 10 °C min^{-1}, respectively.

Preparation of MoP bulk. In a typical procedure, 0.353 g of $(NH_4)_6Mo_7O_{24}\cdot4H_2O$ and 0.264 g of $(NH_4)_2HPO_4$, and 20 ml DI water were added into a 50 ml three-necked flask, which was kept and stirred at 85 °C for 20 h and further dried at 120 °C overnight. The dried sample was grinded and calcined at 500 °C for 10 h in a muffle furnace. Subsequently, the calcined sample was maintained at 850 °C for 2 h with a heating rate of 5 °C min^{-1} under the protection of a H_2/Ar atmosphere (volume ratio: 1:9). The MoP bulk was got after cooling to room temperature.

Preparation of ink. The graphene oxide (GO) was synthesized according to the previously reported methods.[1] 3D-printed inks were prepared by utilizing "evaporation concentration" method. In detail, 500 mg of MoP@NC-1 and 200 mg of carbon nanotube (CNT) were firstly mixed into 10 mL of deionized water (DW) under ultrasonication. Then, 10 mL of GO solution (30 mg mL^{-1}) was homogeneously added into the MoP@NC/CNT solution to form the MoP@NC/CNT/GO solution. Finally, the above MoP@NC-1/CNT/GO solution was kept stirring to evaporate excessive water and achieve a slurry with high viscosity, which was denoted as MoP@NC-1/CNT/GO ink. Additionally, the homogeneous active carbon (AC)/CNT/GO ink was prepared

through adopting the same procedures as the MoP@NC-1/CNT/GO ink, which was produced by using 600 mg of AC and 200 mg of CNT in 10 mL DI water, and 10 mL GO solution (20 mg mL^{-1}) instead.

3D Printing process. The 3D printing was conducted by using a multi-axis printing/dispensing system (RZC-30WK) accompanied by 3D programming software of Auto (Computer Aided Design) CAD. The as-prepared MoP@NC-1/CNT/GO and AC/CNT/GO inks were separately filled into two syringes with metal needles. The woodpile structures were printed on a polytetrafluoroethylene (PTFE) substrate with a preset line spacing of 0.5 mm. The diameter for the nozzle was 200 μm. The printing speeds of the MoP@NC-1/CNT/GO and AC/CNT/GO inks were 2 mm s^{-1}. The height of the nozzle was kept at 0.15 mm to ensure the moderate adhesion of the ink on the PTFE substrate and the adjacent printed layers.

Construction of Potassium-Ion Hybrid Capacitors. 3D-printed MoP@NC-1 and AC electrode were freeze-dried to remove the water solvent. Then, a reduction process of GO was conducted with the aid of hydrogen iodide (HI) vapor.[2] Before the assembly of 3D-printed potassium-ion hybrid capacitors (3DP-PIHCs), the MoP@NC-1 electrode immerse closely with a K foil in the electrolyte for 60 s. 3DP-PIHCs were assembled into the coin cell prototype (CR2032) with a 3D-printed MoP@NC-1 electrode, a 3D-printed AC electrode, and a Whatman GF/F glass membrane serving as anode, cathode, and separator, respectively. The electrolyte used was 0.8 M KPF_6 in ethylene carbon (EC)/diethyl carbonate (DEC) (v:v, 1:1).

Characterizations. X-Ray diffraction (XRD) were recorded on a D8 ADVANCE X-ray diffractometer with Cu Ka radiation (λ =1.5406 Å). The transmission electron microscopy (TEM) and high-resolution transmission electron microscopy (HRTEM) images were observed by FEI TalosF200S. The scanning electron microscopy (SEM) was conducted by7500F, JEOL. X-ray photoelectron spectra (XPS) analyses were made with an AXIS Supra device. The curve fitting of all XPS spectra were accomplished using XPS Peak 4.1 software. All XPS spectra were corrected according to the C 1s line at 284.8 eV. Atomic force microscopy (AFM) images were obtained with a Bruker Dimension FastScan™. The Surface area and pore volume were measured by N_2 adsorption isotherm using an ASAP 2460 Micromeritics instrument by Brunauer–Emmett–Teller (BET) method. Thermogravimetric analysis (TGA, Pyris 1 TGA, Perkin Elmer) was performed in air at a heating rate of 10 °C min^{-1}

Electrochemical measurements. Electrochemical experiments were performed using

two-electrode CR2032 coin cells. In the half-cell test, the electrodes for various MoP@NC composites and MoP bulk were fabricated by coating their corresponding slurries (containing 70 wt% of active materials, 20 wt% of carbon black and 10 wt% of Carboxymethyl Cellulose sodium (CMC) binder) on a copper foil. Potassium metal foils were used as both counter and reference electrodes. The glass fiber membrane (Whatman, GF/D) was used as a separator, and the electrolyte solution was formed as 0.8 M KPF_6 in ethylene carbon (EC)/diethyl carbonate (DEC) (v:v, 1:1). The coin cells were assembled and disassembled in an argon-filled glovebox (O_2 < 0.1 ppm and H_2O < 0.1 ppm). The potassium ion half-cells were galvanostatically charged and discharged at different current densities within a voltage range of 0.01-3.0 V, by using LAND-CT2001A battery-testing instrument under room temperature. Cyclic voltammogram (CV) curves were scanned in a voltage window of 0.01-3.0 V with a scan rate of 1 mV s^{-1}. The electrochemical impedance spectroscopy (EIS) analysis was measured with a frequency range from 100 kHz to 10 mHz by an electrochemical workstation (CHI660E). For the full-cell, activated carbon (AC, kurary Co.) was used as the cathode, which was made by coating their corresponding slurries (containing 80 wt% of AC, 10 wt% of Super P and 10 wt% of Polyvinylidene fluoride (PVDF) binder) onto an aluminum foil.

The energy and power densities of 3DP-PIHCs were calculated by numerically integrating the galvanostatic discharge profiles using the equations below:

$$\mathrm{E} = \int_{t1}^{t2} IV\,dt = \Delta V \times \frac{I}{m} \times t = \frac{(V_{max} + V_{min})}{2} \times \frac{I}{m} \times t \quad (1)$$

$$\mathrm{P} = \mathrm{E}/\mathrm{t} \quad (2)$$

Where t_1 and t_2 are the start time and end time in the discharge process, I is the charge/discharge current, V_{max} and V_{min} are the start and end voltage of the discharge process, and t corresponds to the discharge time.

Computational Methods. The density functional theory (DFT) calculations were carried out using the Vienna ab initio simulation package (VASP) with the projector augmented wave (PAW)[3] pseudopotentials and the function of Perdew, Burke, and Ernzerhof (PBE)[4] based on the generalized gradient approximation (GGA). A cutoff energy of 400 eV was used for the plane-wave basis set. The Brillouin zone was sampled on the basis of the Monkhorst–Pack scheme[5] with a 3 × 3 × 1 k-point mesh. A 7 × 7 graphene supercell with a vacuum region of 15 Å was adopted. The convergence criteria of self-consistent field (SCF) for electronic relaxation and force for atomic relaxation were 1×10^{-5} eV and 0.01 eV $Å^{-1}$, respectively. The binding energy was defined as $E_{ad} = E_{sub+ion} - E_{sub} - E_{ion}$, where $E_{sub+ion}$, E_{sub}, and E_{ion} were the total energies of substrates with the electrolyte ions, clean substrates, and the isolated electrolyte ions, respectively.

Figures section

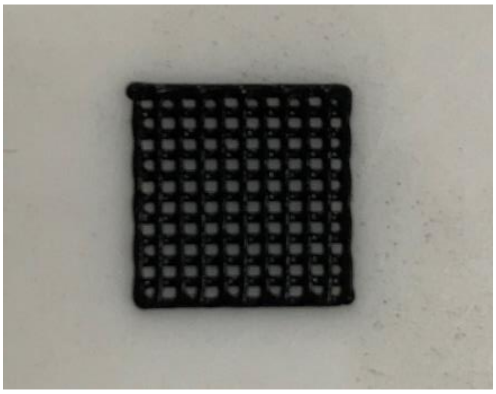

Figure S1. The digital photo of 3D-printed electrode.

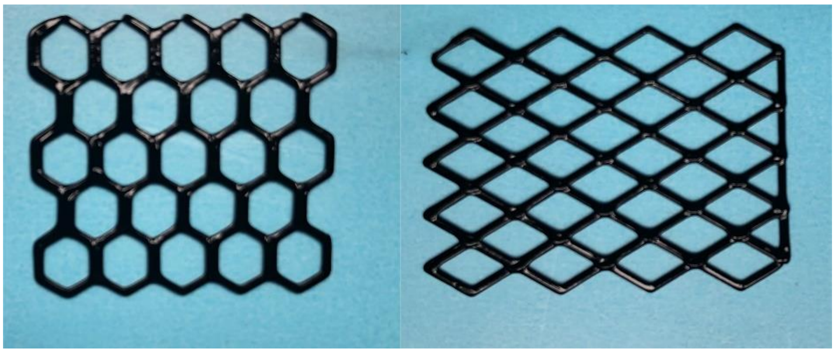

Figure S2. The photographs of 3D-printed shape in different geometric patterns.

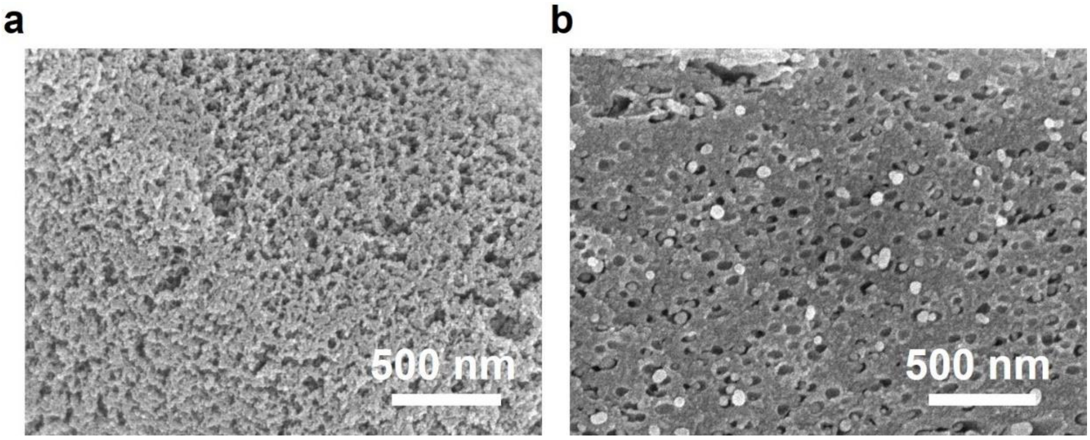

Figure S3. SEM images of (a) MoP@NC-2 and (b) MoP@NC-3.

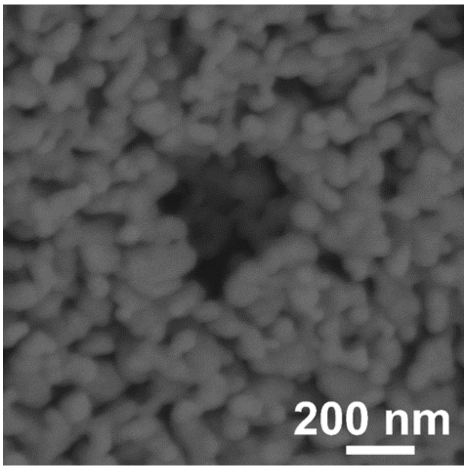

Figure S4. SEM image of MoP bulk.

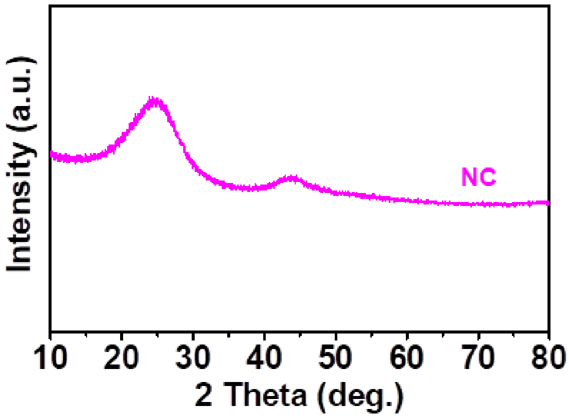

Figure S5. XRD pattern of NC.

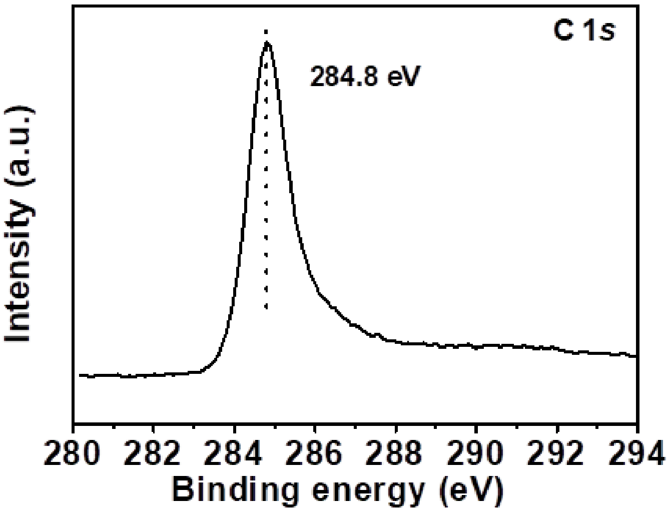

Figure S6. High resolution spectrum of C 1*s* XPS peak in MoP@NC-1.

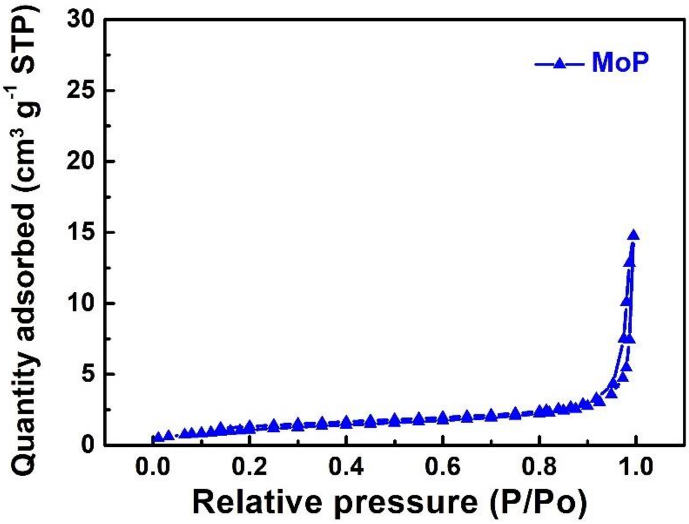

Figure S7. Nitrogen adsorption/desorption isotherm of MoP bulk.

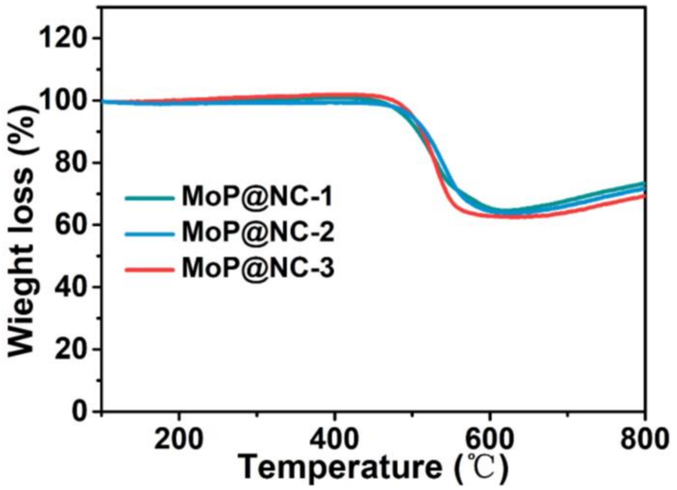

Figure S8. The TGA curves of MoP@NC-1/2/3 composites.

The MoP content was determined by thermogravimetric analysis (TGA) in air atmosphere as shown in Figure S8. According to the equation: $2MoP + 5O_2 \rightarrow 2MoOPO_4$. The MoP transforms to $MoOPO_4$ after heating to 800 °C.[6,7] Consequently, the weight of residues obtained from MoP@NC-1/2/3 composites disclosed that the contents of MoP are about 49.2%, 48.2%, and 46.6% for MoP@NC-1, MoP@NC-2, and MoP@NC-3, respectively.

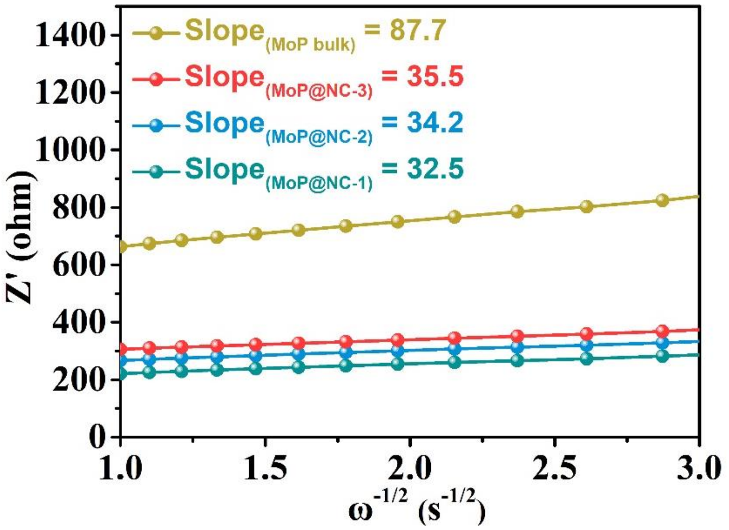

Figure S9. The liner relation of $\omega^{1/2}$ vs. -Z' of MoP@NC-1/2/3 and MoP bulk.

The EIS was employed to evaluate the electrochemical resistance during the discharge/charge process. The diffusion coefficient of K^+ (D_{K+}) can reveal the kinetics process according to the low frequency of EIS curves, which is related to the $\sigma^{-0.5}$. It can be explained by the equation as follows:

$D_{K+} = 0.5(RT/AF^2Cn^2\sigma)^2$

where R is the gas constant (8.314 J mol^{-1} K^{-1}), T is Kelvin temperature (293.15 K), A is the area of electrode, F is the Faraday constant (96485, C mol^{-1}), C is the concentration of potassium ion and n is the electronic transfer number. The σ could be obtained from the slope of −Im(Z) versus $\omega^{-1/2}$, and ω is the angular frequency. The σ values of MoP@NC-1, MoP@NC-2, MoP@NC-3, and MoP bulk are 32.5, 34.2, 35.5, and 87.7, respectively, manifesting the K^+ diffusion of MoP@NC-1 is higher than that of other samples (especially for MoP bulk).[8,9]

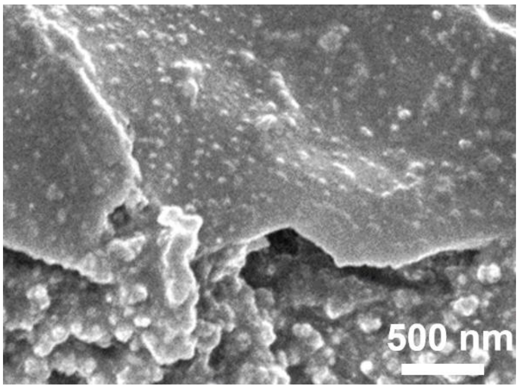

Figure S10. The SEM of the MoP@NC-1 composite after 100 cycling at 1 A g^{-1}.

To get a better understanding of the structural stability, the SEM image of the MoP@NC-1 composite was displayed in Figure S10. Under the aegis and confined effect from the NC skeleton, MoP@NC-1 effectively maintain the original structure,

indicating that the NC skeleton can capture the decomposed crystalline grains, further confirming the advantages of N-doped carbon skeleton.

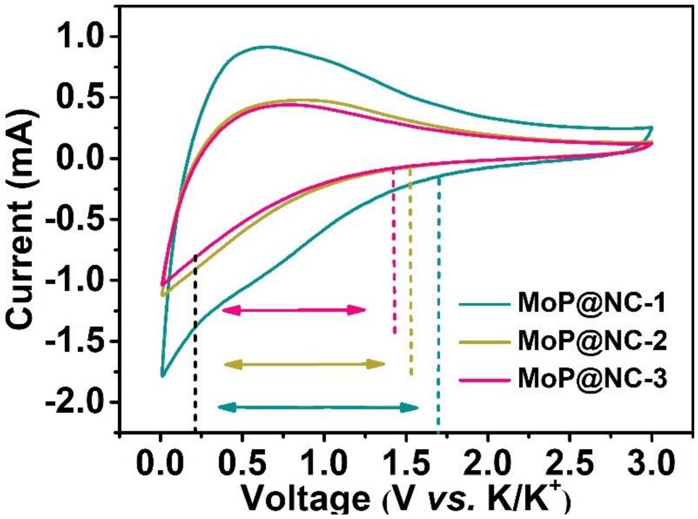

Figure S11. The CV curves of MoP@NC-1, MoP@NC-2, and MoP@NC-3 in a potassium half-cell at scan rate of 2 mV s^{-1}.

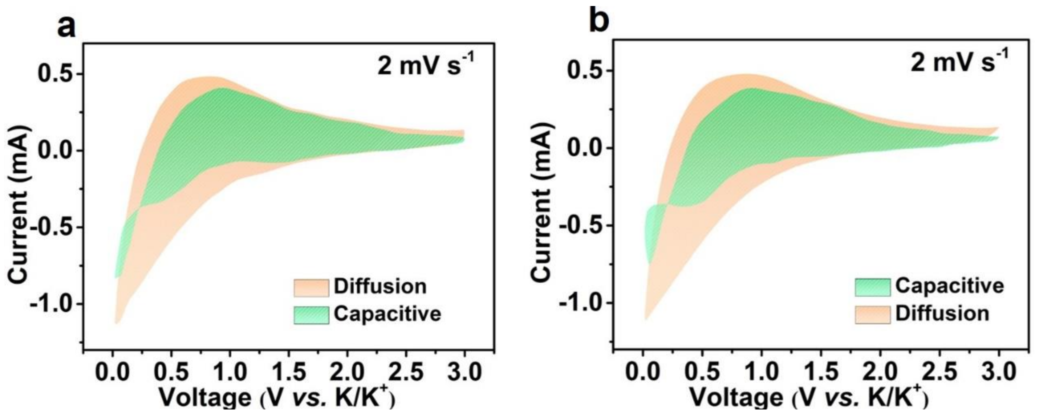

Figure S12. The capacitive contribution (green) and the diffusion contribution (orange) of (a) MoP@NC-2 and (b) MoP@NC-3.

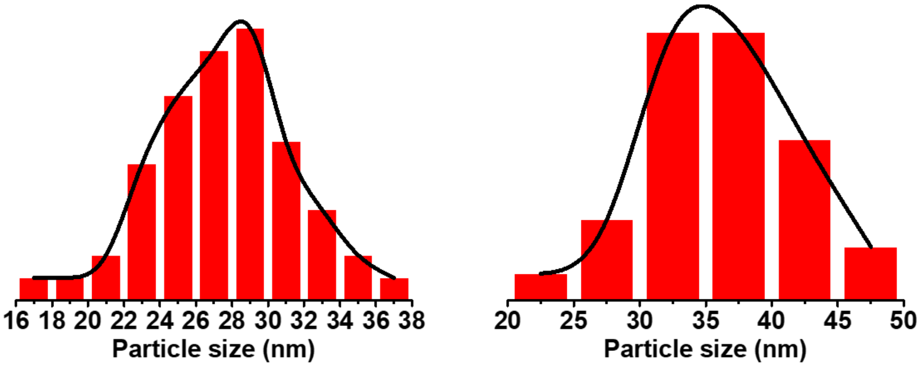

Figure S13. The size distribution quantifications of MoP@NC-2 and MoP@NC-3.

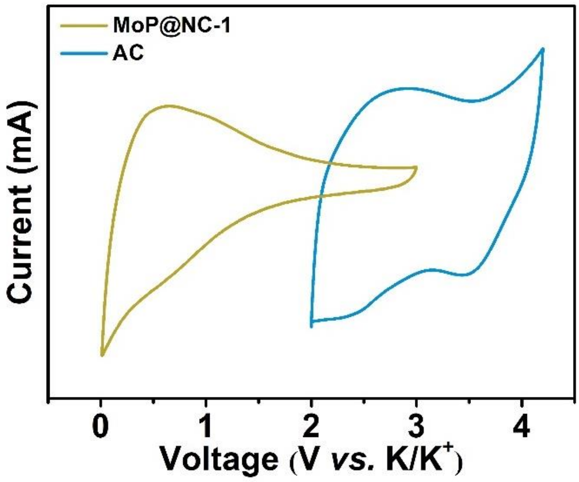

Figure S14. The CV curves of MoP@NC-1 and AC in a potassium half-cell at scan rate of 2 mV s^{-1}.

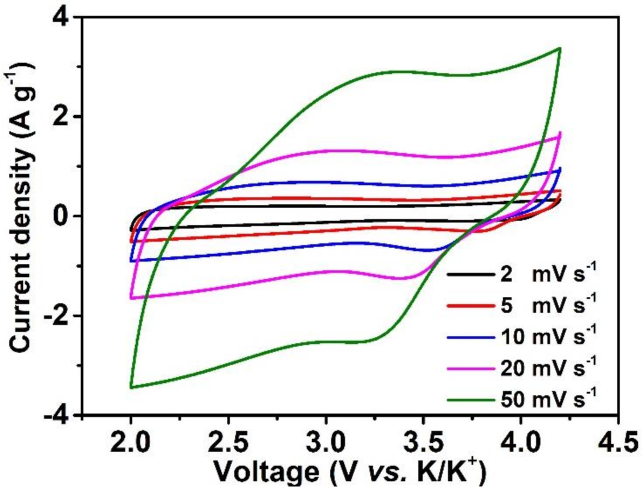

Figure S15. The CV curves of 3D-printed AC electrode in various scan rates.

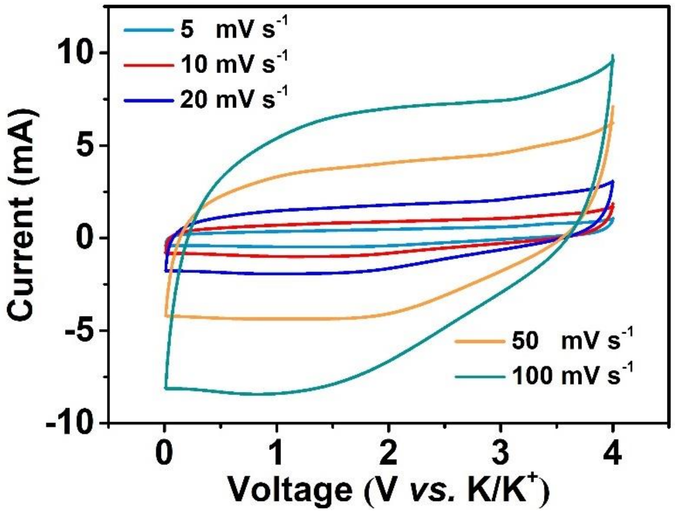

Figure S16. CV curves at different scan rates of MoP@NC-1//AC 3DP-PIHC device.

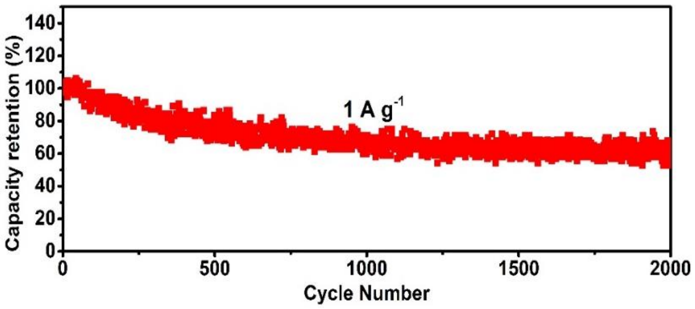

Figure S17. The long cycling performance of the MoP@NC-1//AC 3DP-PIHCs.

The MoP@NC-1//AC 3DP-PIHCs also present good cyclic stability with a capacity retention of 61.2 % over 2000 cycles at 1 A g^{-1} (Figure S17).

Table S1. The comparison of transition metal phosphides for potassium-ion battery anodes.

**Materials**	**Rate capacity**	**Current density [mA g^{-1}]**	**Cycles**	**Capacity [mAh g^{-1}]**	**Voltag [V]**	
CoP⊂NPPCS	2 A g^{-1}<br>54 mAh g^{-1}	100	1000	127	0.01-3	10
MoP@NPCNFs	2A g^{-1}<br>223 mAh g^{-1}	100	200	280	0.01-3	11
FeP@CNBs	2 A g^{-1}<br>37 mAh g^{-1}	100	300	205	0.01-2.5	12
AC@CoP/NCNTs/CNFs	3.2 A g^{-1}<br>292 mAh g^{-1}	800	1000	250	0.01-3	13
Fe-Ni phosphide	5 A g^{-1}<br>38 mAh g^{-1}	200	700	60	0.01-2.5	14
NeCNF@FeP	0.8 A g^{-1}<br>103 mAh g^{-1}	100	1000	210	0.01-3	15

References

[1] Y. Li, T. Gao, Z. Yang, C. Chen, W. Luo, J. Song, E. Hitz, C. Jia, Y. Zhou, B. Liu, *Adv. Mater.* **2017**, *29*, 1700981.

[2] I. K. Moon, J. Lee, R. S. Ruoff, H. Lee, *Nat. Commun.* **2010**, *1*, 1.

[3] P. E. Blochl, *Phys. Rev. B* **1994**, *50*, 17953.

[4] J. P. Perdew, K. Burke, M. Ernzerhof, *Phys. Rev. Lett.* **1996**, *77*, 3865.

[5] H. J. Monkhorst, J. D. Pack, *Phys. Rev. B* **1976**, *13*, 5188.

[6] Z. Huang, H. Hou, C. Wang, S. Li, Y. Zhang, X. Ji, *Chem. Mater.* **2017**, *29*, 7313.

[7] Y. Cao, B. Zhang, X. Ou, Y. Li, C. Wang, L. Cao, C. Peng, J. Zhang, *New J. Chem.* **2019**, *43*, 7386.

[8] S. Li, P. Ge, F. Jiang, H. Shuai, W. Xu, Y. Jiang, Y. Zhang, J. Hu, H. Hou, X. Ji, *Energy Storage Mater.* **2019**, *16*, 267.

[9] P. Ge, H. Hou, S. Li, L. Yang, X. Ji, *Adv. Funct. Mater.* **2018**, *28*, 1801765.

[10] J. Bai, B. Xi, H. Mao, Y. Lin, X. Ma, J. Feng, S. Xiong, *Adv. Mater.* **2018**, *30*, 1802310.

[11] Z. Yi, Y. Liu, Y. Li, L. Zhou, Z. Wang, J. Zhang, H. Cheng, Z. Lu, *Small*, **2020**, *16*, 1905301.

[12] F. Yang, H. Gao, J. Hao, S. Zhang, P. Li, Y. Liu, J. Chen, Z. Guo, *Adv. Funct. Mater.* **2019**, *29*, 1808291.

[13] W. F. Miao, X. Y. Zhao, R. Wang, Y. Q. Liu, L. Li, Z. S, Zhang, W. M. Zhang, *J. Colloid Interface Sci.* **2019**, *556*, 432.

[14] Y. Wu, Y. Yi, Z. Sun, H. Sun, T. Guo, M. Zhang, L. Cui, K. Jiang, Y. Peng, J. Sun, *Chem. Eng. J.* **2020**, *390*, 124515.

[15] X. J. Wang, J. Y. Ma, J. M. Wang, X. F. Li, *J. Alloy. Compd.* **2020**, *821*, 153268.
